# Treatment Effect of Long-Term Antipsychotics on Default-Mode Network Dysfunction in Drug-Naïve Patients With First-Episode Schizophrenia: A Longitudinal Study

**DOI:** 10.3389/fphar.2022.833518

**Published:** 2022-05-20

**Authors:** Mengjie Deng, Zhening Liu, Yanyu Shen, Hengyi Cao, Manqi Zhang, Chang Xi, Wen Zhang, Wenjian Tan, Jinqiang Zhang, Eric Chen, Edwin Lee, Weidan Pu

**Affiliations:** ^1^ Medical Psychological Center, The Second Xiangya Hospital, Central South University, Changsha, China; ^2^ Department of Psychiatry, The Second Xiangya Hospital, Central South University, Changsha, China; ^3^ Mental Health Institute of Central South University, Changsha, China; ^4^ China National Clinical Research Center for Mental Health Disorders, Changsha, China; ^5^ The First Clinical Medical College, Lanzhou University, Lanzhou, China; ^6^ Center for Psychiatric Neuroscience, Feinstein Institute for Medical Research, Hempstead, NY, United States; ^7^ Division of Psychiatry Research, Zucker Hillside Hospital, Glen Oaks, NY, United States; ^8^ School of Psychology, Center for Studies of Psychological Application, South China Normal University, Guangzhou, China; ^9^ Department of Clinical Psychology, The Third Xiangya Hospital, Central South University, Changsha, China; ^10^ Department of Psychiatry, The University of Hong Kong, Pok Fu Lam, Hong Kong SAR, China; ^11^ College of Mechatronics and Automation, National University of Defense Technology, Changsha, China

**Keywords:** default-mode network, external attention system, functional connectivity, first-episode schizophrenia, long-term antipsychotic treatment

## Abstract

**Background:** The maintenance of antipsychotic treatment is an efficient way to prevent the relapse of schizophrenia (SCZ). Previous studies have identified beneficial effects of antipsychotics on brain structural and functional abnormalities during mostly the acute phase in SCZ, but seldom is known about the effects of long-term antipsychotics on the brain. The present study focused on the long-term antipsychotic effect on the default mode network (DMN) dysfunction in SCZ.

**Methods:** A longitudinal study of the functional connectivity (FC) of 11 DMN subdivisions was conducted in 86 drug-naive first-episode patients with SCZ at the baseline and after a long-term atypical antipsychotic treatment (more than 6 months) based on the resting-state functional magnetic resonance image. In total, 52 patients completed the follow-up of clinical and neuroimaging investigations.

**Results:** At the baseline, relative to healthy controls, altered connectivities within the DMN and between the DMN and the external attention system (EAS) were observed in patients. After treatment, along with significant relief of symptoms, most FC alterations between the DMN and the EAS at the baseline were improved after treatment, although the rehabilitation of FC within the DMN was only observed at the link between the posterior cingulate cortex and precuneus. Greater reductions in negative and positive symptoms were both related to the changes of DMN-EAS FC in patients.

**Conclusion:** Our findings provide evidence that maintenance antipsychotics on SCZ is beneficial for the improvement of DMN-EAS competitive imbalance, which may partly contribute to the efficient relapse prevention of this severe mental disorder.

## Introduction

Schizophrenia is a severe and chronic mental disorder with a high relapse rate (81.9%) and functional decline following illness progression (Kissling, 1991; Van Os and Kapur, 2009). Antipsychotic treatment is the major therapy for this severe mental disorder, and at least a 6- to 24-month antipsychotic treatment is recommended in most global clinical guidelines to patients with first-episode schizophrenia (FES) for an optimal response and prevention of relapse (Lehman et al., 2004; Buchanan et al., 2010; Galletly et al., 2016; Crockford, 2017). A most recent meta-analysis demonstrated that compared to short-term interval treatment, longer antipsychotic treatment for up to 24 months significantly prevented the relapse of FES patients (Kishi et al., 2019). However, while many neuroimaging studies have been focused on a short-term (6–12 weeks) antipsychotic effect on the brain structural (Zeng et al., 2016; Kraguljac et al., 2019) and functional (Zong et al., 2019; Duan et al., 2020b) abnormalities in schizophrenia, little is known regarding the efficacy of long-term antipsychotic treatment on brain abnormalities. The answer to this question is important since it may explain why maintenance antipsychotic treatment helps preventing patients from relapse and provides new clues to the drug development for this severe mental disorder.

Schizophrenia is associated with functional dysconnectivity across large-scale brain networks, in which the default-mode network (DMN) is regarded as one of the most important systems implicated in the neuropathology of schizophrenia (Fitzsimmons et al., 2013; Dong et al., 2018). Dysfunction of the DMN has been consistently observed in different clinical samples of schizophrenia patients including first-episode patients (Duan et al., 2015), chronic patients (Alonso-Solis et al., 2015), unaffected siblings (Liu et al., 2012), and individuals at high risk (Wang et al., 2015) and is also related to cognitive deficits and positive symptoms such as delusion and hallucinations (Whitfield-Gabrieli et al., 2009). Furthermore, given the evidence that the DMN is an important information integration hub in the brain (Buckner et al., 2008), a large number of studies have investigated the changes of the DMN interactions with other core networks such as the external attention system (EAS, including a fronto-parietal network, a dorsal attention network, and a salience network) (Palaniyappan and Liddle, 2012; O’Neill et al., 2019; Supekar et al., 2019). Importantly, the disrupted interactions across large-scale networks are shown to be related to clinical symptoms and cognitive deficits in schizophrenia (Frith, 1995; Peled, 1999). An intriguing question thereby raised on clinical practice is whether the current pharmacological treatment could retrieve the DMN function in schizophrenia.

During the past 2 decades, increasing neuroimaging studies have paid intense attention on the short-term antipsychotic (6–8 weeks) effect on the DMN connectivity. Several studies identified beneficial effects of antipsychotics on the DMN dysfunction (Zong et al., 2019; Duan et al., 2020a; Duan et al., 2020b), but others reported null findings (Sambataro et al., 2010; Kraguljac et al., 2016a). The conflicting findings may relate to the short durations of treatment within 8 weeks, which may be insufficient to reach treatment stability (Lehman et al., 2004). How longer pharmacological treatment would affect the function of DMN in this disorder is unclear.

To date, only four studies have investigated the efficacy of antipsychotic treatments with a long duration (more than 6 months) on the brain dysfunction in schizophrenia (Li et al., 2016; Guo et al., 2017; Guo et al., 2018; Li et al., 2018). Three studies reported a significant therapeutic effect on the DMN dysfunction (Li et al., 2016; Guo et al., 2017; Guo et al., 2018), although none of them performed corrections on the group comparison of the functional magnetic resonance imaging (fMRI) data at the voxel level. The relatively low statistic power in these studies is due to small samples, which calls for the long-time follow-up of drug-naïve FES with larger samples. Moreover, none of the previous studies have explored the antipsychotic effect on the functional integrations between the DMN and other core networks, which is key to the understanding of antipsychotic effects on the DMN dysfunction in schizophrenia.

The present study focused on the therapeutic effect of long-term antipsychotic treatment on the DMN-relevant dysfunction in drug-naïve FES schizophrenia. According to the recommendation of most clinical guidelines that 6–24 months of antipsychotics is effective for an optimal response and prevention of relapse (Lehman et al., 2004; Buchanan et al., 2010; Galletly et al., 2016; Crockford, 2017), we followed 86 drug-naïve FES patients with 6- to 24-month treatment at two time points in a real-world setting. There were three aims in the present study: 1) To investigate the dysfunction within DMN regions as well as the dysfunctional integration between the DMN and other core brain networks in drug-naïve FES, 2) to examine long-term antipsychotic effects on the DMN dysfunction in patients, and 3) to assess the relationship between the DMN functional changes and symptom retrieval.

## Materials and Methods

### Participants

A total of 246 participants (86 patients with FES) were recruited from three clinical centers: 1) The Second Xiangya Hospital (Changsha, Hunan Province, China, Dataset #1 and Dataset #2), 2) The First Affiliated Hospital of Zhejiang University (Hangzhou, Zhejiang Province, China, Dataset #3), and 3) Queen Mary Hospital, The University of Hong Kong (Hong Kong, China, Dataset #4). The details of each dataset are present in Supplementary Table S1, and the inclusion and exclusion criteria of patients are given in Supplementary Methods S1. Patients with FES were recruited using the Structured Clinical Interview of the DSM-IV-patient version (SCID-P). Following prior studies (Anticevic et al., 2015b; Wu et al., 2021; Zhang et al., 2021), the data site was later included as a covariate into further fMRI imaging data analysis. The clinical symptom severity of patients was evaluated by qualified psychiatrists using the Scale for the Assessment of Positive Symptoms (SAPS) (Andreasen et al., 1995) and the Scale for the Assessment of Negative Symptoms (SANS) (Andreasen, 1989). All participants completed clinical and resting-state fMRI measurements at the baseline, and 52 patients completed the follow-up investigation when they continued treatment between 6 and 24 months. Medication was monitored every 2 months through face-to-face interviews or telephone tracing by researchers. Patients who conducted subsequent visits to the hospital on time every 2 months underwent face-to-face interviews. However, some patients did not live in the local areas, so it was much more convenient for them to complete the medication monitoring by phone contact. Furthermore, for the patients who were monitored by telephone, we also attempted to contact the patients’ families or guardians who monitored their medication treatment. During the medication monitoring process, we asked questions to ensure their compliance for treatment, such as the medication dosage every day, the manifestation, and the side effects of the medication. In total, 34 patients were dropped out at follow-up, out of which 19 patients lost contact or quit this research and 15 patients could not maintain the compliance or quit the antipsychotic treatment. No significant difference in clinical symptom scores at the baseline was found between the dropped-out patients and follow-up patients (Supplementary Table S2). Notably, all patients were treated with a second-generation antipsychotic drug, and the dosages were determined by the treating psychiatrists (Supplementary Methods S2). The medication doses for each patient were converted to chlorpromazine equivalence (50–1,000 mg/day).

A total of 160 age- and sex-matched healthy controls were recruited from the local community and screened using the SCID non-patient edition. None of the healthy controls had a first-degree relative with psychiatric disorders. The healthy controls were scanned once after they were recruited. All the participants signed voluntary informed consent. The procedure and consent forms were reviewed and approved by the local ethics committee at each data collection site.

### Image Data Acquisition and Preprocessing

All participants underwent the resting-state fMRI scanning. Participants were required to remain motionless and awake with their eyes closed. Soft earplugs and foam pads were used to decrease scanner noise and head motion. The imaging parameters of each clinical center were set the same at both scans (baseline and follow-up). Statistical Parametric Mapping 12 (SPM12, http://www.fil.ion.ucl.ac.uk/spm) and Data Processing Assistant for Resting-State fMRI (DPABI, http://www.rfmri.org/) (Yan et al., 2016) were used for further image preprocessing. The details of the imaging parameters of each clinical center and preprocessing procedures are provided in the Supplementary Material. To ensure data quality, participants were excluded if their head motion was more than 3.0 mm or 3.0° during the resting-state fMRI, which included 11 patients at the baseline, five patients at follow-up, and three at healthy controls. Finally, a total of 232 participants (75 patients with FES) at the baseline and 47 patients at follow-up were selected for the following analysis in the present study. Furthermore, head motion scrubbing regression was performed to eliminate the confounding effect of subtle head movements (for details on the “scrubbing” procedure, please see Supplementary Methods S3).

### Functional Connectivity

Functional connectivity (FC) was conducted based on a seed-based correlation approach (Horwitz et al., 1998). To examine the DMN dysfunction, a fine-grained DMN template developed by Andrews-Hanna et al. (2010) was applied to calculate the FC between 11 DMN subregions and the rest of the brain in a voxel-wise manner. This template suggests that the DMN was composed of two midline cores and two distinct subsystems with strong intrinsic correlations. The midline cores include the posterior cingulate cortex (PCC) and the anterior medial prefrontal cortex (aMPFC). The subsystems are composed of the dorsal medial prefrontal cortex (dMPFC) subsystem and the medial temporal lobe (MTL) subsystem. The former subsystem includes the dMPFC, the temporoparietal junction (TPJ), the lateral temporal cortex (LTC), and the temporal pole (TempP), and the latter subsystem includes the ventral medial prefrontal cortex (vMPFC), the posterior inferior parietal lobule (pIPL), the retrosplenial cortex (Rsp), the parahippocampal cortex (PHC), and hippocampal formation (HF^+^). To improve normality, Fisher r-to-z transformations were applied to the resulting correlation maps.

### Statistical Analysis

The statistical analysis of fMRI data was conducted using the SPM12 software. To compare the differences of functional connectivity of 11 DMN subregions with other regions across the whole brain, the two-sample *t*-test was performed between patients (*n* = 75) and healthy controls (*n* = 157), with age, sex, education, data site, and frame-wise displacement (FD) values (see Supplementary Method S3) as covariates. Then, by using the brain regions with FC alterations at the baseline for each DMN subregion as mask, the paired *t*-test was performed to identify the longitudinal changes of FC in patients (*n* = 47) between the baseline (T1) and after treatment (T2). The significance level for the aforementioned comparisons was set at *p* < 0.05 under false discovery rate (FDR) correction at the voxel level.

To compare the differences of socio-demographic and clinical data between groups, the two sample *t*-test was used for continuous variables, while the Chi-square test was used for categorical variables. Pearson’s correlation was assessed to explore the correlations between the FC changes (FC values at T2 minus FC values at T1) and clinical symptom improvement (symptom score at T1 minus symptom score at T2) in patients. The statistical analysis of socio-demographic and clinical data as well as Pearson’s correlation was conducted using SPSS 19.0. The statistical threshold was set at *p* < 0.05.

## Results

### Demographic and Clinical Characteristics

As shown in Table 1, at the baseline, there were no significant differences between patients and healthy controls in age and sex (all *p* > 0.05), except for education (*p* < 0.001). After treatment, the SAPS and SANS total scores were significantly decreased (SAPS: *p* < 0.001; SANS: *p* < 0.01). Of note, 42 FES patients showed more than 30% reduction of positive symptoms and 31 patients showed more than 30% reduction of negative symptoms (Supplementary Table S3).

**TABLE 1:** Demographic data and clinical data of participants.

Characteristic	FES at the baseline *N* = 75	Healthy controls *N* = 157	Statistics (*t*/*χ* ^2^)
Age (years)	22.28 ± 5.99	22.77 ± 4.94	−0.62
Sex (male: female)	31: 44	70: 87	0.22
Education (years)	12.15 ± 2.97	13.62 ± 2.73	−3.73***
Duration of illness (months)	5.64 ± 4.46	—	
DUP (months)	5.22 ± 4.33	—	
Clinical symptom scores			
SAPS total scores	30.71 ± 17.31	—	
SANS total scores	27.93 ± 20.56	—	
**Characteristic**	**FES at follow-up *N* = 47**		**Statistics (*t*/*χ* ^2^)**
	**T1**	**T2**	
Duration of treatment (months)	—	15.04 ± 8.01	
CPZ equivalents (mg)	—	292.03 ± 202.57	
Clinical symptom scores			
SAPS total scores	33.54 ± 18.68	8.45 ± 10.13	^a^9.40***
SANS total scores	29.71 ± 19.69	19.18 ± 17.35	^a^3.38**

FES, first-episode schizophrenia patients; T1, time point at the baseline; T2, time point after treatment; DUP, duration of untreated psychosis; CPZ, chlorpromazine; SAPS, Scale for the Assessment of Positive Symptoms; SANS, Scale for the Assessment of Negative Symptoms.

a Paired *t*-test in patients with first-episode schizophrenia between the baseline and follow-up.

***p < 0.001; **p < 0.01.

### Abnormal Functional Connectivity of the Default Mode Network in Drug-Naïve First-Episode Schizophrenia Patients at the Baseline

**FIGURE 1:**
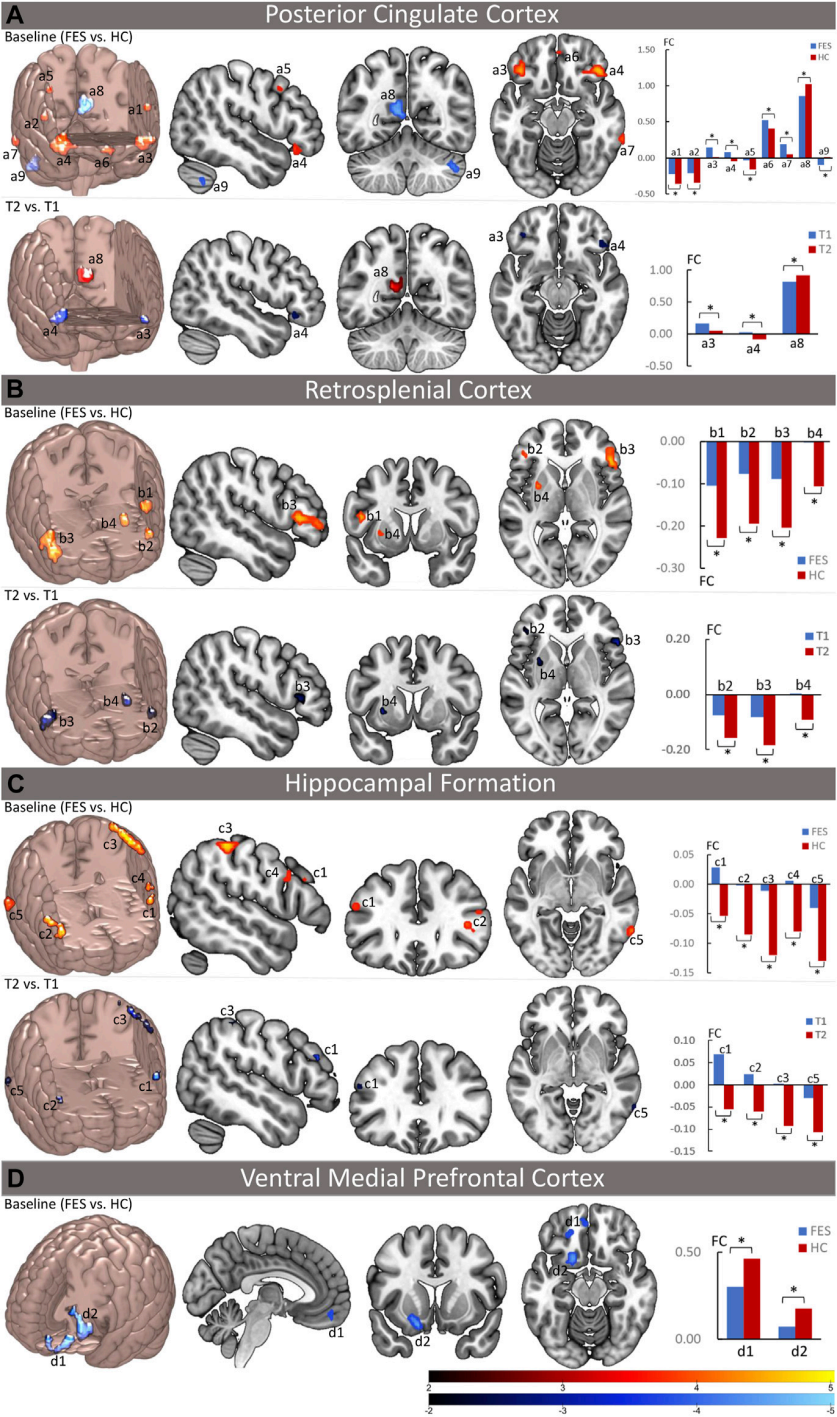
Abnormal functional connectivity of the DMN at the baseline and the effect of long-term antipsychotic treatments on the DMN function in FES patients. The left upper part of **(A–C)** and the left part of **(D)** in each panel depicts the brain regions with significantly increased (red) and/or decreased (blue) functional connectivity (FC) with DMN seed regions (the posterior cingulate cortex, the retrosplenial cortex, hippocampal formation, and the ventral medial prefrontal cortex) at the baseline in FES patients compared to healthy controls (*p* < 0.05, corrected by FDR), while the bar charts in the right upper part of **(A–C)** and the right part of **(D)** show the mean values of the increased (red) and/or decreased (blue) FC with DMN seed regions at the baseline in patients and healthy controls. The left lower part brain maps in **(A–C)** depict the brain regions with significant FC changes with DMN seed regions after treatment in FES patients compared to their baseline (T2 vs. T1, *p* < 0.05, corrected by FDR), while the bar charts in the right lower part of **(A–C)** show the mean values of the FC with significant changes after treatment at both the baseline and follow-up time points. FES, first-episode schizophrenia patients; HC, healthy controls; T1, time point at the baseline; T2, time point after treatment; FC, functional connectivity values; *p < 0.05 (corrected by FDR); a1 = opercular part of the left inferior frontal gyrus; a2 = opercular part of the right inferior frontal gyrus; a3 = orbital part of the left inferior frontal gyrus; a4 = orbital part of the right inferior frontal gyrus; a5 = right middle frontal gyrus; a6 = left rectus gyrus; a7 = right inferior temporal gyrus; a8 = left precuneus; a9 = right cerebellum; b1 = opercular part of the left inferior frontal gyrus; b2 = triangular part of the left inferior frontal gyrus; b3 = triangular part of the right inferior frontal gyrus; b4 = left putamen; c1 = triangular part of the left inferior frontal gyrus; c2 = triangular part of the right inferior frontal gyrus; c3 = left inferior parietal lobule; c4 = left precentral gyrus; c5 = right inferior temporal gyrus; d1 = left anterior medial prefrontal gyrus; d2 = left ventral caudal medial prefrontal gyrus.

As for the two midline cores of the DMN, the PCC, but not the aMPFC, showed significant FC alterations with the EAS and other DMN subregions in patients compared to HCs. For the two DMN subsystems, the MTL subsystem (including the vMPFC, Rsp, and HF^+^), but not the dMPFC subsystem, showed significant FC alterations with the EAS and other DMN sub-regions in patients (Figure 1; Supplementary Table S4). Specifically, the PCC showed increased FC (decreased anticorrelation) with the EAS regions such as the bilateral inferior frontal gyrus (IFG, including the opercular part and orbital part) and the right middle frontal gyrus (MFG); meanwhile, within the DMN itself, the PCC exhibited increased FC with the left rectus and the right inferior temporal gyrus (ITG) and decreased FC with the left precuneus in FES patients. In the MTL subsystem, the Rsp showed increased FC (decreased anticorrelation) with the EAS regions such as the IFG (including the left opercular part and bilateral triangular part) and the left putamen, while the HF^+^ showed increased FC (decreased anticorrelation) with the EAS regions such as the bilateral IFG (triangular part), left inferior parietal lobule (IPL), left precentral gyrus, and right ITG. Moreover, the vMPFC showed decreased FC within the DMN regions including aMPFC and ventral caudal MPFC (vcMPFC).

### Effect of the Antipsychotic Treatment on the Default Mode Network Function in First-Episode Schizophrenia Patients

After treatment, as the clinical symptoms improved, the alterations of FC in the DMN at the baseline including the PCC and MTL subsystems in patients were significantly ameliorated (Figure 1; Table 2). For the PCC, the increased FC with the bilateral IFG (orbital part) at the baseline was decreased, whereas the decreased FC with the left precuneus at the baseline was increased in patients after treatment. For the MTL subsystem, the increased FC of Rsp with the bilateral IFG (triangular part) and the left putamen at the baseline was decreased, while the increased FC of HF^+^ with the bilateral IFG (triangular part), the left IPL, and the right ITG at the baseline was decreased in patients as well after treatment. However, the decreased FC of the vMPFC with the aMPFC and vcMPFC had no significant changes after treatment.

**TABLE 2:** Effects of long-term atypical antipsychotic treatment on DMN functions in FES.

Brain region	MNI			*T* value	Voxels
	x	y	z		
ROI: Posterior cingulate cortex					
T2 vs. T1					
ORBinf.L	−39	42	−15	−3.24	6
ORBinf.R	39	33	−6	−3.37	32
PCUN.L	−15	−57	15	4.00	36
ROI: Retrosplenial cortex					
T2 vs. T1					
IFGtriang.L	−42	36	3	−2.66	10
IFGtriang.R	54	21	−3	−3.87	27
PUT.L	−30	6	0	−3.38	10
ROI: Hippocampal formation					
T2 vs. T1					
IFGtriang.L	−51	30	24	−4.05	6
IFGtriang.R	42	27	15	−2.91	6
IPL.L	−63	−36	39	−3.11	52
ITG.R	63	−60	−3	−2.80	10

ROI, regions of interest (DMN, subregions); MNI, Montreal Neurological Institute coordinate; T1, time point at the baseline; T2, time point after treatment; ORBinf.L, orbital part of the left inferior frontal gyrus; ORBinf.R, orbital part of the right inferior frontal gyrus; PCUN.L, left precuneus; IFGtriang.L, triangular part of the left inferior frontal gyrus; IFGtriang.R, triangular part of the right inferior frontal gyrus; PUT.L, left putamen; IPL.L, left inferior parietal lobule; ITG.R, right inferior temporal gyrus.

To validate our findings, three supplementary analyses were further performed on the neuroimaging data. First, due to this, 42 out of the 47 followed-up patients showed clinically significant symptom reduction (more than 30% reduction on SAPS). We contrasted the DMN FC map only in these 42 patients between follow-up and the baseline (T2 vs. T1) and found very similar results to the original findings (Supplementary Figure S1; Supplementary Table S5). Second, by dividing our patients into two groups according to their duration of treatment (DoT): one group with DoT less than 1 year (*n* = 18) and the other group with DoT more than 1 year (*n* = 29), we compared the DMN FC maps in each group between follow-up and the baseline (T2 vs. T1), respectively, and showed that only the group with DoT more than 1 year exhibited significant alleviation of the DMN dysfunction (Supplementary Figure S2; Supplementary Table S6). However, two DoT groups showed no significant differences at either the baseline or follow-up time point. Third, the sample included five patients with age < 16 years old; to control for the possible neurodevelopment on our findings, we excluded the patients with age <16 years old and repeated our analysis, which also found very similar results compared to our original findings (Supplementary Figure S3; Supplementary Tables S7, S8).

### Associations of the Default Mode Network Functional Improvement With Clinical Symptom Relief in First-Episode Schizophrenia Patients

**FIGURE 2:**
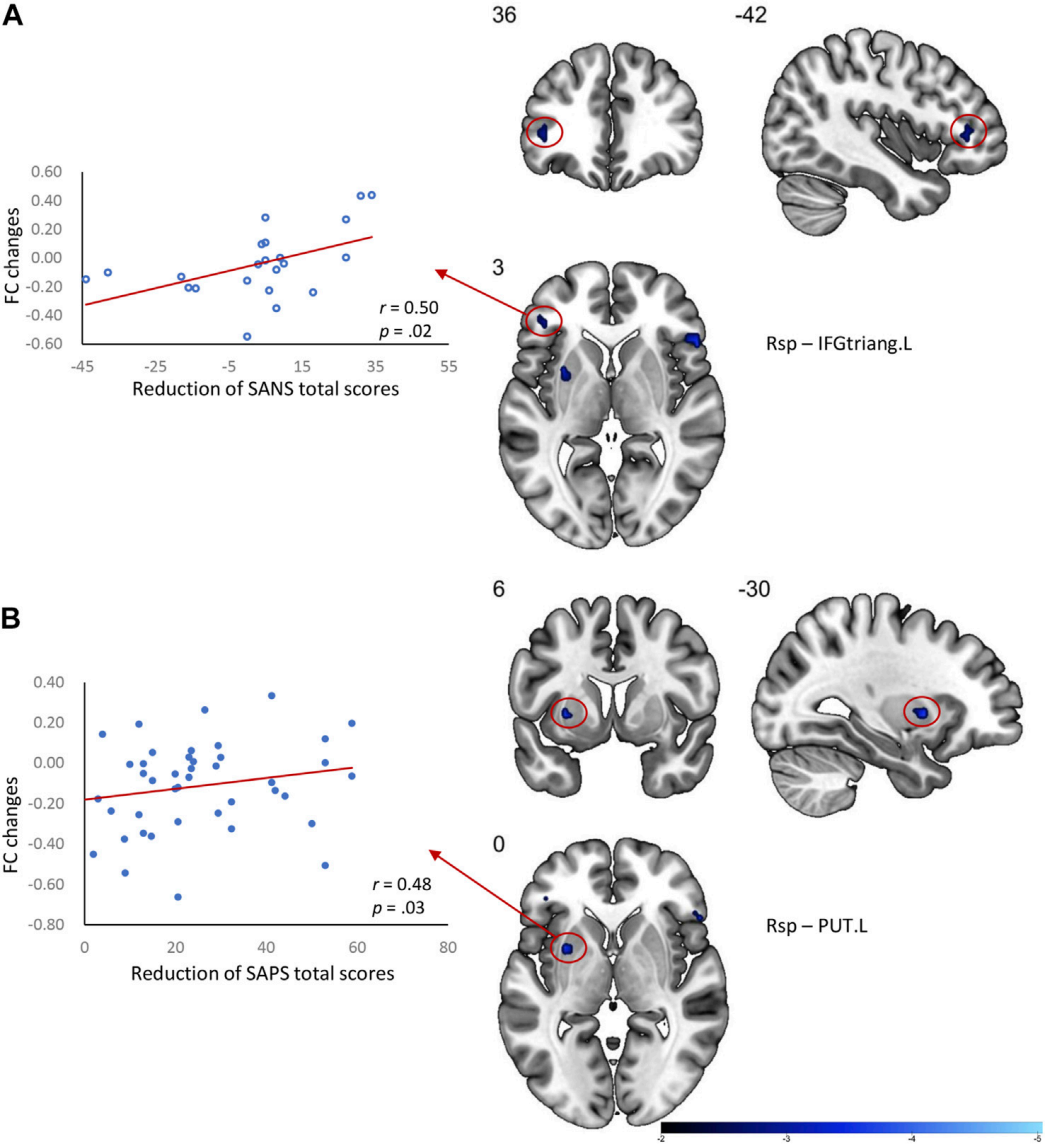
Correlation between the DMN FC changes and clinical symptom relief in FES patients after treatment. **(A)** Greater changes of FC between the Rsp and IFGtriang.L related to a better improvement of the SANS total score in patients; **(B)** Greater changes of FC between the Rsp and PUT.L related to better improvement of SAPS total score in patients. Rsp, retrosplenial cortex; IFGtriang.L, triangular part of the left inferior frontal gyrus; PUT.L, left putamen; FC, functional connectivity values; SANS, scale for the assessment of negative symptoms; SAPS, scale for the assessment of positive symptoms.

The long-term antipsychotic treatment in our cohort of drug-naïve FES patients resulted in an overall reduction in clinical symptoms measured by the SANS and SAPS. As the clinical symptoms improved, we also observed that the DMN dysfunctions were ameliorated after treatment. Thus, subsequent Pearson’s correlation analyses were performed to examine the associations between the reduction of clinical symptoms and the changes in FC of the DMN regions in patients (Figure 2). A greater reduction of the SAPS total score was positively related to the change of the FC in the Rsp with the left putamen (*r* = 0.48, uncorrected *p* = 0.03), while greater reduction of the SANS total score (*r* = 0.50, uncorrected *p* = 0.02) was positively related to the change of the FC in the Rsp with the left IFG (triangular part).

## Discussion

In this longitudinal resting-state fMRI study on drug-naïve patients with FES, by using a fine-grained DMN template containing two midline cores and two subsystems, we mapped the whole brain FC of the DMN and examined the long-term (more than 6 months) atypical antipsychotic treatment effect on the DMN-relevant dysfunction. Consistent with prior studies (Kraguljac et al., 2016b; Dong et al., 2018; Duan et al., 2020a), at the baseline, we found that the DMN exhibited abnormal FC in the posterior midline core (e.g., PCC) and the medial temporal subsystem (including vMPFC, Rsp, and HF^+^) in FES patients. Of note, the DMN showed abnormal FC not only within the DMN itself (i.e., between PCC and precuneus/ITG/rectus and between vMPFC and aMPFC/vcMPFC) but also with other core intrinsic brain networks such as the EAS (i.e., between PCC and IFG, between Rsp and IFG, and between HF^+^ and IFG). Clinical symptoms including positive and negative symptoms, measured by the SAPS and SANS, were significantly relieved in patients after treatment. Meanwhile, most FC alterations between the DMN and the EAS were significantly improved after treatment, although the functional relief within the DMN itself was only observed at the link between the PCC and precuneus. Furthermore, greater reduction of the negative symptoms related to the changes of DMN-EAS functional integration (i.e., FC between the Rsp and IFG), while greater reduction of the positive symptoms related to the changes of FC in the Rsp with the putamen in FES patients. The present study extends prior evidence that antipsychotics not only have a beneficial effect on the DMN-related dysfunction in schizophrenia at the acute phase (Zong et al., 2019; Duan et al., 2020a) but also hold the therapeutic effect through maintenance treatment. Most importantly, our findings support that long-term atypical antipsychotic therapy alleviates the negative symptoms in schizophrenia, which may be partly contributed by the improvement of the DMN-EAS integration after longer medication therapy observed in the present study.

In agreement with prior studies (Garrity et al., 2007; Buckner et al., 2008), the present study found the abnormality of spontaneous activity in the posterior midline core (the PCC) and the MTL subsystem. The PCC with distributed anatomical and functional connections throughout the cortex has been consistently identified as the “hub” of the brain (Andrews-Hanna et al., 2010) and correlated with internal mentations such as autobiography memory, theory of mind, and self-referential functions (Buckner et al., 2008). In line with its “hub” role, we found a widespread abnormal connectivity of the PCC in drug-naïve FES patients within the DMN and with other core intrinsic networks, suggesting that the integrative functions of this hub are impaired in drug-naïve FES patients. Moreover, the MTL subsystem, critical for recalling the past and imagination of the future (Andrews-Hanna et al., 2010), was broadly disrupted at the vMPFC, Rsp, and HF^+^ in this study. Interestingly, as observed in the present study, while the FC of the vMPFC was impaired within the DMN (the aMPFC and vcMPFC), the Rsp and HF^+^ were impaired in communicating with the other core networks such as the EAS (including the IFG, IPL, and putamen). These findings together suggest that the DMN dysfunction in schizophrenia was not limited to the within-DMN impairment but was extended to its integration with other brain networks.

After treatment, we observed that the positive and negative symptoms were alleviated in patients. Of importance, the DMN-relevant dysfunctions were as well ameliorated as the clinical symptoms improved. A novel finding in the present study is that the long-term antipsychotic treatment seems to target mainly on the abnormalities of integration between the DMN and the EAS. For both the PCC and the MTL subsystems, most of the reduced anticorrelations (negative FC) with the EAS subregions (i.e., IFG, IPL, ITG, etc.) at the baseline were increased after treatment, suggesting that the competitive imbalance between the DMN and the EAS consistently observed in prior schizophrenia studies (Whitfield-Gabrieli et al., 2009; Pu et al., 2016) is modulated by a longer antipsychotic treatment.

Mounting evidence has supported that the greater anticorrelation between the DMN and the EAS facilitates the attention to be shifted from introspective mental activity to extrospective goal-directed processing (Fox et al., 2009; Sala-Llonch et al., 2012) and promotes the adaptive and efficient behaviors in healthy subjects (Eichele et al., 2008). In schizophrenia, such a competitive imbalance between the DMN and the EAS has been associated with various clinical symptoms and cognitive deficits (Pu et al., 2016). A recent short-term longitudinal fMRI study using the dynamic FC approach found that an abnormal connection of the dorsal anterior insula (a key node of the salience network) with the precuneus (a key node of the DMN) was normalized by an 8-week risperidone treatment in drug-naïve FES patients (Duan et al., 2020b). Our findings extend the evidence by showing that a longer antipsychotic treatment has more extensive therapeutic effects on the DMN-EAS imbalance, which may provide a possible explanation on why maintenance treatments efficiently prevent a relapse of schizophrenia. Consistent with this notion, our correlation analysis found that a greater increase of the anticorrelation between the Rsp and IFG related to an improvement of the negative symptoms, which is one of the most unrelenting symptoms in schizophrenia.

We also noted that the anticorrelation between the Rsp and putamen was reduced at the baseline and increased after treatment. Of note, a greater increase of the anticorrelation in this link related to the improvement of positive symptoms in the patients. Putamen is a subregion of the striatum rich in dopamine D_2_ receptors and consistently involved in the pathology of schizophrenia (Simpson et al., 2010), particularly for the positive symptoms including delusion and hallucinations (Liu et al., 2019). Prior follow-up studies also identified the short-term antipsychotic effect on the functional impairments of this subcortical region and its specific relationship with an alleviation of positive symptoms in schizophrenia (Sarpal et al., 2015; Wu et al., 2019). Our findings extend the literature showing that long-term antipsychotic treatment has a maintenance effect on the functional disruption in the putamen.

Within the DMN, only the decreased PCC-PCUN FC at the baseline was increased after treatment, suggesting that the within-DMN dysfunction is modulated by the current pharmacological treatment only to a certain extent. Similar to our findings, a recent 1-year follow-up fMRI study found no changes of the vMPFC and precuneus after treatment in schizophrenia patients (Li et al., 2016). Moreover, previous short-term longitudinal studies in schizophrenia showed mixed findings of antipsychotic effects on the within-DMN dysfunction (Kraguljac et al., 2016b; Zong et al., 2019). These findings suggest that neither short-term nor long-term antipsychotic treatment has an efficient effect on the within-DMN dysfunction in schizophrenia, which may provide a potential target for new drug development in the future.

Atypical antipsychotics exert a major neurochemical influence on the dopamine pathway and mainly act on D_2–4_ dopamine receptors and 5-HT_2A_ receptors in cortical regions, such as the DMN regions (Kapur and Seeman, 2001). Previous evidence has documented a significant correlation of FC measures with dopamine and serotonin signaling (Tan et al., 2012; Tollens et al., 2018). Thus, the relief of the DMN-EAS imbalance and within-DMN dysfunction after treatment in the present study are possibly attributed to dopamine and serotonin antagonistic action of the atypical antipsychotics. Moreover, Anticivic et al. (2015a) revealed a potential association between the glutamate metabolism and the DMN activation in normal participants, and they further evidenced that the blockade of the N-methyl-D-aspartate (NMDA) receptor led to psychotic-like symptoms and relevant DMN-EAS imbalance in normal subjects (Anticevic et al., 2012). These findings, together with the evidence that antipsychotics also change other neurotransmitters including glutamate, glutamine, and GABA (Tan et al., 2012; Tollens et al., 2018), suggest another possibility that the relief of the DMN dysfunction in the present study relies on the modulation of the glutamate system.

The present study aimed to examine the effect of long-term antipsychotic treatments on DMN abnormalities in a real-world setting. Given that most clinical guidelines recommend 6–24 months of psychotic treatment on patients with first-episode schizophrenia for an optimal response and prevention of relapse (Lehman et al., 2004; Buchanan et al., 2010; Galletly et al., 2016; Crockford, 2017), this study followed the patients with the DoT between 6 and 24 months. However, whether DoT influences the fMRI signal remains hitherto unknown owing to very few long-term follow-up studies existing in the literature. To control the potential confounding effect of DoT, we divided the patients into one group with DoT less than 1 year (*n* = 18) and another group with DoT more than 1 year (*n* = 29) and found a significant alleviation of the DMN dysfunction only in the group with DoT more than 1 year. One of the possible explanations for this finding is that only an antipsychotic treatment longer than 1 year is efficient for alleviating the DMN dysfunctions in schizophrenia. However, two groups showed no significant differences either at the baseline or at the follow-up time point, suggesting that the relatively smaller sample of the group with DoT less than 1 year may limit the statistic power for a significant discovery of the drug effect. It calls for a future study with a larger sample following patients at multiple time points to clarify this issue.

## Limitations

Several limitations should be noted in the present study. First, the education between patients and healthy controls was not matched, although the following analyses included education as one of the covariates. Second, FDR correction was performed for group comparisons at the voxel level to control for type I errors in the fMRI data. However, for the secondary analysis involving 11 DMN subdivisions, we cannot exclude the possibility of a type I error. We urge caution when interpreting the subregional findings. Third, the range of the treatment duration in follow-up patients was not standardized. On one hand, although the longitudinal design with sampling of the standardized time point is optimal to model the characteristics of the brain network changes during treatment in schizophrenia, it is very difficult and resource-intensive to collect the follow-up data from the same schizophrenia patient during the long-term treatment with a sufficient sample size. On the other hand, after an acute treatment (4–8 weeks), the majority of schizophrenia patients will be relatively stable on their symptoms, and most clinical guidelines suggest a 6–24 months maintenance treatment for the first-episode patient for an optimal response and prevention of relapse. Thus, this study, under a real-world setting, recruited schizophrenia patients with a treatment duration between 6 and 24 months to explore the maintenance or long-term effects of antipsychotic treatment on the DMN dysfunction. Future studies with more elegant designs to standardize the long-term follow-up time point would help to better understand the antipsychotic effect on the brain networks in this severe mental disorder. Finally, all the correlation findings did not survive after FDR correction, possibly due to our relatively small sample. However, it is proposed that the usual trajectory with rapid symptom reduction in the early treatment of schizophrenia patients may obscure the correlation between fMRI signals and clinical symptoms. Future follow-up studies with larger samples need to replicate our findings.

## Conclusion

In summary, the present study documented that long-term antipsychotic treatments had beneficial effects on the DMN dysfunction in schizophrenia. Our findings suggested that the DMN-EAS competitive imbalance might be the main target of long-term antipsychotics, while the within-DMN dysfunction could be relieved only to a certain extent by current pharmacological treatment. Furthermore, the improvement of the DMN-EAS imbalance was related to the relief of both positive and negative symptoms in schizophrenia patients, suggesting that the effectiveness of long-term antipsychotics on clinical symptoms possibly relied on its modulation on the DMN-EAS integration. Our findings may provide a novel clue to understand why maintenance antipsychotics efficiently prevent relapse of schizophrenia and strengthen the direction of new drug development on the within-DMN dysfunction for this severe mental disorder.

## Data Availability

The datasets presented in this article are not readily available because the datasets used in this research are not public and are only available among the institutions that collected the data in this study. Requests to access the datasets should be directed to Weidan Pu, weidanpu@csu.edu.cn.

## References

[B1] Alonso-Solís A. Vives-Gilabert Y. Grasa E. Portella M. J. Rabella M. Sauras R. B. (2015). Resting-state Functional Connectivity Alterations in the Default Network of Schizophrenia Patients with Persistent Auditory Verbal Hallucinations. Schizophr Res. 161 (2-3), 261–268. 10.1016/j.schres.2014.10.047 25468173

[B2] Andreasen N. C. Arndt D. Flaum M. Nopoulos P. (1995). Correlational Studies of the Scale for the Assessment of Negative Symptoms and the Scale for the Assessment of Positive Symptoms: An Overview and Update. Psychopathology 28, 7–17. 10.1159/000284894 7871123

[B3] Andreasen N. C. (1989). The Scale for the Assessment of Negative Symptoms (SANS): Conceptual and Theoretical Foundations. Br. J. Psychiatry Suppl. 7, 49–58. 10.1192/s0007125000291496 2695141

[B4] Andrews-Hanna J. R. Reidler J. S. Sepulcre J. Poulin R. Buckner R. L. (2010). Functional-Anatomic Fractionation of the Brain's Default Network. Neuron 65 (4), 550–562. 10.1016/j.neuron.2010.02.005 20188659 PMC2848443

[B5] Anticevic A. Corlett P. R. Cole M. W. Savic A. Gancsos M. Tang Y. (2015a). N-methyl-D-aspartate Receptor Antagonist Effects on Prefrontal Cortical Connectivity Better Model Early Than Chronic Schizophrenia. Biol. Psychiatry 77 (6), 569–580. 10.1016/j.biopsych.2014.07.022 25281999

[B6] Anticevic A. Gancsos M. Murray J. D. Repovs G. Driesen N. R. Ennis D. J. (2012). NMDA Receptor Function in Large-Scale Anticorrelated Neural Systems with Implications for Cognition and Schizophrenia. Proc. Natl. Acad. Sci. U S A. 109 (41), 16720–16725. 10.1073/pnas.1208494109 23012427 PMC3478611

[B7] Anticevic A. Haut K. Murray J. D. Repovs G. Yang G. J. Diehl C. (2015b). Association of Thalamic Dysconnectivity and Conversion to Psychosis in Youth and Young Adults at Elevated Clinical Risk. JAMA Psychiatry 72 (9), 882–891. 10.1001/jamapsychiatry.2015.0566 26267151 PMC4892891

[B8] Buchanan R. W. Kreyenbuhl J. Kelly D. L. Noel J. M. Boggs D. L. Fischer B. A. (2010). The 2009 Schizophrenia PORT Psychopharmacological Treatment Recommendations and Summary Statements. Schizophr Bull. 36 (1), 71–93. 10.1093/schbul/sbp116 19955390 PMC2800144

[B9] Buckner R. L. Andrews-Hanna J. R. Schacter D. L. (2008). The Brain's Default Network: Anatomy, Function, and Relevance to Disease. Ann. N. Y Acad. Sci. 1124, 1–38. 10.1196/annals.1440.011 18400922

[B10] Crockford D. A. D. Addington D. (2017). Canadian Schizophrenia Guidelines: Schizophrenia and Other Psychotic Disorders with Coexisting Substance Use Disorders. Can. J. Psychiatry 62, 624–634. 10.1177/0706743717720196 28886671 PMC5593250

[B11] Dong D. Wang Y. Chang X. Luo C. Yao D. (2018). Dysfunction of Large-Scale Brain Networks in Schizophrenia: A Meta-Analysis of Resting-State Functional Connectivity. Schizophr Bull. 44 (1), 168–181. 10.1093/schbul/sbx034 28338943 PMC5767956

[B12] Duan H. F. Gan J. L. Yang J. M. Cheng Z. X. Gao C. Y. Shi Z. J. (2015). A Longitudinal Study on Intrinsic Connectivity of hippocampus Associated with Positive Symptom in First-Episode Schizophrenia. Behav. Brain Res. 283, 78–86. 10.1016/j.bbr.2015.01.022 25619684

[B13] Duan X. Hu M. Huang X. Dong X. Zong X. He C. (2020a). Effects of Risperidone Monotherapy on the Default-Mode Network in Antipsychotic-Naïve First-Episode Schizophrenia: Posteromedial Cortex Heterogeneity and Relationship with the Symptom Improvements. Schizophr Res. 218, 201–208. 10.1016/j.schres.2020.01.001 31954611

[B14] Duan X. Hu M. Huang X. Su C. Zong X. Dong X. (2020b). Effect of Risperidone Monotherapy on Dynamic Functional Connectivity of Insular Subdivisions in Treatment-Naive, First-Episode Schizophrenia. Schizophr Bull. 46 (3), 650–660. 10.1093/schbul/sbz087 31504959 PMC7147596

[B15] Eichele T. Debener S. Calhoun V. D. Specht K. Engel A. K. Hugdahl K. (2008). Prediction of Human Errors by Maladaptive Changes in Event-Related Brain Networks. Proc. Natl. Acad. Sci. U S A. 105 (16), 6173–6178. 10.1073/pnas.0708965105 18427123 PMC2329680

[B16] Fitzsimmons J. Kubicki M. Shenton M. E. (2013). Review of Functional and Anatomical Brain Connectivity Findings in Schizophrenia. Curr. Opin. Psychiatry 26 (2), 172–187. 10.1097/YCO.0b013e32835d9e6a 23324948

[B17] Fox M. D. Zhang D. Snyder A. Z. Raichle M. E. (2009). The Global Signal and Observed Anticorrelated Resting State Brain Networks. J. Neurophysiol. 101 (6), 3270–3283. 10.1152/jn.90777.2008 19339462 PMC2694109

[B18] Frith C. (1995). Functional Imaging and Cognitive Abnormalities. Lancet 346 (8975), 615–620. 10.1016/s0140-6736(95)91441-2 7651009

[B19] Galletly C. Castle D. Dark F. Humberstone V. Jablensky A. Killackey E. (2016). Royal Australian and New Zealand College of Psychiatrists Clinical Practice Guidelines for the Management of Schizophrenia and Related Disorders. Aust. N. Z. J. Psychiatry 50 (5), 410–472. 10.1177/0004867416641195 27106681

[B20] Garrity A. G. Pearlson G. D. McKiernan K. Lloyd D. Kiehl K. A. Calhoun V. D. (2007). Aberrant "default Mode" Functional Connectivity in Schizophrenia. Am. J. Psychiatry 164 (3), 450–457. 10.1176/ajp.2007.164.3.450 17329470

[B21] Guo W. Liu F. Chen J. Wu R. Li L. Zhang Z. (2018). Treatment Effects of Olanzapine on Homotopic Connectivity in Drug-free Schizophrenia at Rest. World J. Biol. Psychiatry 19, S106–S114. 10.1080/15622975.2017.1346280 28649941

[B22] Guo W. Liu F. Chen J. Wu R. Li L. Zhang Z. (2017). Olanzapine Modulation of Long- and Short-Range Functional Connectivity in the Resting Brain in a Sample of Patients with Schizophrenia. Eur. Neuropsychopharmacol. 27 (1), 48–58. 10.1016/j.euroneuro.2016.11.002 27887859

[B23] Horwitz B. Rumsey J. M. Donohue B. C. (1998). Functional Connectivity of the Angular Gyrus in normal reading and Dyslexia. Proc. Natl. Acad. Sci. U S A. 95 (15), 8939–8944. 10.1073/pnas.95.15.8939 9671783 PMC21181

[B24] Kapur S. Seeman P. (2001). Does Fast Dissociation from the Dopamine D(2) Receptor Explain the Action of Atypical Antipsychotics?: A New Hypothesis. Am. J. Psychiatry 158 (3), 360–369. 10.1176/appi.ajp.158.3.360 11229973

[B25] Kishi T. Ikuta T. Matsui Y. Inada K. Matsuda Y. Mishima K. (2019). Effect of Discontinuation V. Maintenance of Antipsychotic Medication on Relapse Rates in Patients with Remitted/Stable First-Episode Psychosis: A Meta-Analysis. Psychol. Med. 49 (5), 772–779. 10.1017/S0033291718001393 29909790

[B26] Kissling W. (1991). The Current Unsatisfactory State of Relapse Prevention in Schizophrenic Psychoses-Ssuggestions for Improvement. Clin. Neuropharmacol 14 (Suppl. 2), S33–S44. 1684309

[B27] Kraguljac N. V. Anthony T. Skidmore F. M. Marstrander J. Morgan C. J. Reid M. A. (2019). Micro- and Macrostructural White Matter Integrity in Never-Treated and Currently Unmedicated Patients with Schizophrenia and Effects of Short-Term Antipsychotic Treatment. Biol. Psychiatry Cogn. Neurosci. Neuroimaging 4 (5), 462–471. 10.1016/j.bpsc.2019.01.002 30852126 PMC6500745

[B28] Kraguljac N. V. White D. M. Hadley J. A. Visscher K. Knight D. ver Hoef L. (2016a). Abnormalities in Large Scale Functional Networks in Unmedicated Patients with Schizophrenia and Effects of Risperidone. Neuroimage Clin. 10, 146–158. 10.1016/j.nicl.2015.11.015 26793436 PMC4683457

[B29] Kraguljac N. V. White D. M. Hadley N. Hadley J. A. ver Hoef L. Davis E. (2016b). Aberrant Hippocampal Connectivity in Unmedicated Patients with Schizophrenia and Effects of Antipsychotic Medication: A Longitudinal Resting State Functional MRI Study. Schizophr Bull. 42 (4), 1046–1055. 10.1093/schbul/sbv228 26873890 PMC4903060

[B30] Lehman A. F. Lieberman J. A. Dixon L. B. McGlashan T. H. Miller A. L. Perkins D. O. (2004). Practice Guideline for the Treatment of Patients with Schizophrenia, Second Edition. Am. J. Psychiatry 161 (2), 1–56. 15000267

[B31] Li F. Lui S. Yao L. Hu J. Lv P. Huang X. (2016). Longitudinal Changes in Resting-State Cerebral Activity in Patients with First-Episode Schizophrenia: A 1-Year Follow-Up Functional MR Imaging Study. Radiology 279 (3), 867–875. 10.1148/radiol.2015151334 27007945

[B32] Li M. Deng W. Das T. Li Y. Zhao L. Ma X. (2018). Neural Substrate of Unrelenting Negative Symptoms in Schizophrenia: A Longitudinal Resting-State fMRI Study. Eur. Arch. Psychiatry Clin. Neurosci. 268 (7), 641–651. 10.1007/s00406-017-0851-5 29128871

[B33] Liu H. Kaneko Y. Ouyang X. Li L. Hao Y. Chen E. Y. (2012). Schizophrenic Patients and Their Unaffected Siblings Share Increased Resting-State Connectivity in the Task-Negative Network but Not its Anticorrelated Task-Positive Network. Schizophr Bull. 38 (2), 285–294. 10.1093/schbul/sbq074 20595202 PMC3283150

[B34] Liu J. Yao L. Zhang W. Deng W. Xiao Y. Li F. (2019). Dissociation of Fractional Anisotropy and Resting-State Functional Connectivity Alterations in Antipsychotic-Naive First-Episode Schizophrenia. Schizophr Res. 204, 230–237. 10.1016/j.schres.2018.08.005 30121186

[B35] O'Neill A. Mechelli A. Bhattacharyya S. (2019). Dysconnectivity of Large-Scale Functional Networks in Early Psychosis: A Meta-Analysis. Schizophr Bull. 45 (3), 579–590. 10.1093/schbul/sby094 29982729 PMC6483589

[B36] Palaniyappan L. Liddle P. F. (2012). Does the Salience Network Play a Cardinal Role in Psychosis? an Emerging Hypothesis of Insular Dysfunction. J. Psychiatry Neurosci. 37 (1), 17–27. 10.1503/jpn.100176 21693094 PMC3244495

[B37] Peled A. (1999). Multiple Constraint Organization in the Brain: A Theory for Schizophrenia. Brain Res. Bull. 49 (4), 245–250. 10.1016/s0361-9230(99)00048-9 10424844

[B38] Pu W. Luo Q. Palaniyappan L. Xue Z. Yao S. Feng J. (2016). Failed Cooperative, but Not Competitive, Interaction between Large-Scale Brain Networks Impairs Working Memory in Schizophrenia. Psychol. Med. 46 (6), 1211–1224. 10.1017/S0033291715002755 26743997

[B39] Sala-Llonch R. Peña-Gómez C. Arenaza-Urquijo E. M. Vidal-Piñeiro D. Bargalló N. Junqué C. (2012). Brain Connectivity during Resting State and Subsequent Working Memory Task Predicts Behavioural Performance. Cortex 48 (9), 1187–1196. 10.1016/j.cortex.2011.07.006 21872853

[B40] Sambataro F. Blasi G. Fazio L. Caforio G. Taurisano P. Romano R. (2010). Treatment with Olanzapine Is Associated with Modulation of the Default Mode Network in Patients with Schizophrenia. Neuropsychopharmacology 35 (4), 904–912. 10.1038/npp.2009.192 19956088 PMC3055362

[B41] Sarpal D. K. Robinson D. G. Lencz T. Argyelan M. Ikuta T. Karlsgodt K. (2015). Antipsychotic Treatment and Functional Connectivity of the Striatum in First-Episode Schizophrenia. JAMA Psychiatry 72 (1), 5–13. 10.1001/jamapsychiatry.2014.1734 25372846 PMC4286512

[B42] Simpson E. H. Kellendonk C. Kandel E. (2010). A Possible Role for the Striatum in the Pathogenesis of the Cognitive Symptoms of Schizophrenia. Neuron 65 (5), 585–596. 10.1016/j.neuron.2010.02.014 20223196 PMC4929859

[B43] Supekar K. Cai W. Krishnadas R. Palaniyappan L. Menon V. (2019). Dysregulated Brain Dynamics in a Triple-Network Saliency Model of Schizophrenia and its Relation to Psychosis. Biol. Psychiatry 85 (1), 60–69. 10.1016/j.biopsych.2018.07.020 30177256

[B44] Tan H. Y. Chen A. G. Kolachana B. Apud J. A. Mattay V. S. Callicott J. H. (2012). Effective Connectivity of AKT1-Mediated Dopaminergic Working Memory Networks and Pharmacogenetics of Anti-dopaminergic Treatment. Brain 135 (Pt 5), 1436–1445. 10.1093/brain/aws068 22525159 PMC3338927

[B45] Tollens F. Gass N. Becker R. Schwarz A. J. Risterucci C. Künnecke B. (2018). The Affinity of Antipsychotic Drugs to Dopamine and Serotonin 5-HT2 Receptors Determines Their Effects on Prefrontal-Striatal Functional Connectivity. Eur. Neuropsychopharmacol. 28 (9), 1035–1046. 10.1016/j.euroneuro.2018.05.016 30006253

[B46] van Os J. Kapur S. (2009). Schizophrenia. Lancet 374 (9690), 635–645. 10.1016/S0140-6736(09)60995-8 19700006

[B47] Wang D. Zhou Y. Zhuo C. Qin W. Zhu J. Liu H. (2015). Altered Functional Connectivity of the Cingulate Subregions in Schizophrenia. Transl Psychiatry 5, e575. 10.1038/tp.2015.69 26035059 PMC4490280

[B48] Whitfield-Gabrieli S. Thermenos H. W. Milanovic S. Tsuang M. T. Faraone S. V. McCarley R. W. (2009). Hyperactivity and Hyperconnectivity of the Default Network in Schizophrenia and in First-Degree Relatives of Persons with Schizophrenia. Proc. Natl. Acad. Sci. U S A. 106 (4), 1279–1284. 10.1073/pnas.0809141106 19164577 PMC2633557

[B49] Wu G. Palaniyappan L. Zhang M. Yang J. Xi C. Liu Z. (2021). Imbalance between Prefronto-Thalamic and Sensorimotor-Thalamic Circuitries Associated with Working Memory Deficit in Schizophrenia. Schizophr Bull. 48, 251–261. 10.1093/schbul/sbab086 PMC8781324 34337670

[B50] Wu R. Ou Y. Liu F. Chen J. Li H. Zhao J. (2019). Reduced Brain Activity in the Right Putamen as an Early Predictor for Treatment Response in Drug-Naive, First-Episode Schizophrenia. Front. Psychiatry 10, 741. 10.3389/fpsyt.2019.00741 31649567 PMC6791918

[B51] Yan C. G. Wang X. D. Zuo X. N. Zang Y. F. (2016). DPABI: Data Processing & Analysis for (Resting-State) Brain Imaging. Neuroinformatics 14 (3), 339–351. 10.1007/s12021-016-9299-4 27075850

[B52] Zeng B. Ardekani B. A. Tang Y. Zhang T. Zhao S. Cui H. (2016). Abnormal white Matter Microstructure in Drug-Naive First Episode Schizophrenia Patients before and after Eight Weeks of Antipsychotic Treatment. Schizophr Res. 172 (1-3), 1–8. 10.1016/j.schres.2016.01.051 26852402

[B53] Zhang M. Palaniyappan L. Deng M. Zhang W. Pan Y. Fan Z. (2021). Abnormal Thalamocortical Circuit in Adolescents with Early-Onset Schizophrenia. J. Am. Acad. Child. Adolesc. Psychiatry 60 (4), 479–489. 10.1016/j.jaac.2020.07.903 32791099

[B54] Zong X. Hu M. Pantazatos S. P. Mann J. J. Wang G. Liao Y. (2019). A Dissociation in Effects of Risperidone Monotherapy on Functional and Anatomical Connectivity within the Default Mode Network. Schizophr Bull. 45 (6), 1309–1318. 10.1093/schbul/sby175 30508134 PMC6811838

