# Supplementary material for: Treatment Effect of Long-Term Antipsychotics on Default-Mode Network Dysfunction in Drug-Naïve Patients With First-Episode Schizophrenia: A Longitudinal Study
Source: Front Pharmacol. 2022 May 20;13:833518. doi: 10.3389/fphar.2022.833518 (PMC9171718; doi:10.3389/fphar.2022.833518)
Supplement: Supplementary file 1 [file DataSheet1.docx]

Supplementary Materials

**Methods**

*S1 Inclusion and exclusion criteria of patients*

The inclusion criteria for first episode schizophrenia (FES) patients were: (1) age between 14-40 years old; (2) Han Chinese ethnicity; (3) 6 years of education or above; (4) right-handed preference; (5) meeting DSM-IV criteria for schizophrenia; (6) first episode (illness duration < 18 months), drug-naïve or having antipsychotics for less than 2 weeks. The exclusion criteria were: (1) substance-related disorder; (2) neurological disorders or severe physical diseases; (3) recorded loss of consciousness (duration > 5 min); (4) any contra-indications to magnetic resonance imaging (MRI) scanning.

*S2 Details of antipsychotics treatment*

Antipsychotics treatment in 52 follow-up FES patients consisted of risperidone (14 patients), paliperidone (10 patients), olanzapine (6 patients), quetiapine (4 patients), ziprasidone (3 patients), aripiprazole (2 patients), amisulpride (2 patient) and combined atypical antipsychotics (11 patients).

*S3 Image data acquisition and preprocessing*

Resting-state functional magnetic resonance imaging (fMRI) images of Dataset 1# was obtained using Philips Gyroscan Achieva 3.0T scanner in the axial direction. Gradient-echo echo-planar imaging sequence was used with the following parameters: TR = 2000 ms, TE = 30 ms, slice thickness = 4 mm, slice gap = 4 mm, acquisition matrix = 64×64, field of view = 240×240 mm^2^, flip angle = 90°, and voxel size = 1.67×1.67×4 mm^3^. Each brain volume comprised 36 slices, and the functional run contained 250 image volumes. Dataset 2# were obtained using Siemens Achieva 3.0T scanner, with Gradient-echo echo-planar imaging sequence using the following parameters: TR = 2000 ms, TE = 30 ms, slice thickness = 4 mm, slice gap = 5 mm, acquisition matrix = 64×64, field of view = 250×250 mm^2^, flip angle = 80°, and voxel size = 4×4×5 mm^3^. Each brain volume comprised 32 slices, and the functional run contained 216 image volumes. Dataset 3# was collected using Philips Gyroscan Achieva 3.0T in the axial direction, with a gradient-echo echo-planar imaging sequence using the following parameters: TR = 2000 ms, TE = 35 ms, slice thickness = 5 mm, slice gap = 6 mm, acquisition matrix = 100×100, field of view = 240×240 mm^2^, flip angle = 80°, and voxel size = 1.67×1.67×6 mm^3^. Each brain volume comprised 24 slices, and the functional run contained 200 image volumes. And Dataset 4# was collected using Philips Gyroscan Achieva 3.0T scanner, with Gradient-echo echo-planar imaging sequence using the following parameters: TR = 2000 ms, TE = 30 ms, slice thickness = 4 mm, slice gap = 4 mm, acquisition matrix = 72×68, field of view = 230×230 mm^2^, flip angle = 90°, and voxel size = 1.6×1.6×4 mm^3^. Each brain volume comprised 32 slices, and the functional run contained 250 image volumes.

The initial 10 timepoints of volumes were discarded for scanner stabilization and for participants’ adaptation to the circumstances. The remaining volumes were corrected for the acquisition delay between slices, followed by a realignment to the middle volume. To ensure data quality, participants were excluded if their head motion were more than 3.0 mm or 3.0° during resting-state fMRI. And totally 11 patients at baseline, 5 patients at follow-up and 3 healthy controls were excluded. Finally, a total of 232 participants (75 patients with FES) at baseline and 47 patients at follow-up were selected for the following analysis in the present study. After realignment, images were then spatially normalized into Montreal Neurological Institute standardized space resampled to 3×3×3 mm^3^ voxels and spatially smoothed with a Gaussian kernel (full width at half-maximum = 8 mm). Linear detrending was conducted and followed by nuisance covariates regression including the signals from cerebrospinal fluid and white matter, 6-parameter rigid-body motion correction parameters as well as the global mean signal. We also performed the head motion scrubbing regression to eliminate the confounding effect of subtle head movement. Following suggestions of a previous study by Power et al. (Power et al., 2012), signal from each ‘bad’ frame defined as frame-wise displacement (FD) > 0.5 and its neighbors (1 frame before and 2 frames after) were flagged for regression. No significant differences on the mean absolute FD were observed between FES patients and healthy controls (HC) (FES: 0.16±0.10, HC: 0.16±0.08, *t* = -0.16, *p* = .87). Subsequently, all time-series was then high- and low-pass filtered at 0.01 Hz and 0.1 Hz, respectively.

**Table S1** Participants composition of datasets from 3 clinical centers

| **Clinical centers** | **Dataset** | **Compositions of participants** | | |
| --- | --- | --- | --- | --- |
|  |  | **FES at baseline** | **HC** | **FES at follow-up** |
| The Second Xiangya Hospital | 1# | 21 | 100 | 17 |
|  | 2# | 8 | 7 | 5 |
| The First Affiliated Hospital of Zhejiang University | 3# | 25 | 50 | 24 |
| Queen Mary Hospital, The University of Hong Kong | 4# | 32 | 0 | 6 |

**Note:** FES, first-episode schizophrenia patients; HC, healthy controls.

**Table S2** Clinical symptoms comparison between dropout and follow-up patients at baseline

| **Clinical symptoms** | **Patients** | | ***t*** | ***p*** |
| --- | --- | --- | --- | --- |
|  | **Dropout** | **Follow-up** |  |  |
| **SAPS** | 27.21±14.14 | 32.79±18.79 | -1.36 | > .05 |
| **SANS** | 25.99±21.76 | 29.08±19.95 | -0.63 | > .05 |

**Note:** SANS, scale for the assessment of negative symptoms; SAPS, scale for the assessment of positive symptoms.

**Table S3** Clinical symptoms score reduction rate in follow-up patients after long-term treatment

| **Clinical symptoms** | **Mean±SD (%)** | **Frequency  (reduction rate≥30%)** |
| --- | --- | --- |
| **SAPS** | 75.04±28.79 | 42 |
| **SANS** | 12.14±108.22 | 31 |

**Note:** SANS, scale for the assessment of negative symptoms; SAPS, scale for the assessment of positive symptoms.

**Table S4** Abnormal functional connectivity of DMN in FES patients at baseline compared to healthy controls

| ROI: Posterior Cingulate Cortex | | | | | | ROI: Retrosplenial Cortex | | | | | |
| --- | --- | --- | --- | --- | --- | --- | --- | --- | --- | --- | --- |
| **Brain region** | **MNI** | | | **T Value** | **Voxels** | **Brain region** | **MNI** | | | **T Value** | **Voxels** |
|  | **x** | **y** | **z** |  |  |  | **x** | **y** | **z** |  |  |
| **FES vs. HC** |  |  |  |  |  | **FES vs. HC** |  |  |  |  |  |
| IFGoperc.L | -54 | 12 | 18 | 3.92 | 6 | IFGoperc.L | -48 | 6 | 18 | 4.57 | 20 |
| IFGoperc.R | 54 | 15 | 15 | 3.88 | 8 | IFGtriang.L | -42 | 36 | 3 | 4.15 | 10 |
| ORBinf.L | -39 | 33 | -12 | 4.60 | 57 | IFGtriang.R | 48 | 27 | 6 | 4.92 | 92 |
| ORBinf.R | 42 | 33 | -15 | 4.67 | 76 | PUT.L | -30 | 3 | 3 | 4.25 | 11 |
| MFG.R | 51 | 15 | 42 | 3.85 | 5 | ROI: Hippocampal Formation | | | | | |
| REC.L | -3 | 48 | -18 | 3.98 | 11 | **Brain region** | **MNI** | | | **T Value** | **Voxels** |
| ITG.R | 69 | -39 | -15 | 4.01 | 12 |  | **x** | **y** | **z** |  |  |
| PCUN.L | -9 | -54 | 15 | -4.70 | 68 | **FES vs. HC** |  |  |  |  |  |
| Cerebellum.R | 45 | -54 | -42 | -4.33 | 25 | IFGtriang.L | -51 | 30 | 27 | 3.81 | 6 |
| ROI: Ventral Medial Prefrontal Cortex | | | | | | IFGtriang.R | 39 | 30 | 12 | 4.16 | 25 |
| **Brain region** | **MNI** | | | **T Value** | **Voxels** | IPL.L | -54 | -36 | 54 | 4.87 | 78 |
|  | **x** | **y** | **z** |  |  | PreCG.L | -54 | 12 | 30 | 3.90 | 5 |
| **FES vs. HC** |  |  |  |  |  | ITG.R | 66 | -57 | -6 | 4.29 | 17 |
| aMPFC.L | -18 | 42 | -21 | -4.05 | 47 |  |  |  |  |  |  |
| vcMPFC.L | -15 | 12 | -18 | -4.78 | 87 |  |  |  |  |  |  |

**Note:** ROI, regions of interest (DMN subregions); MNI, Montreal Neurological Institute coordinate; FES, first-episode schizophrenia patients; HC, healthy controls; IFGoperc.L, opercular part of left inferior frontal gyrus; IFGoperc.R, opercular part of right inferior frontal gyrus; ORBinf.L, orbital part of left inferior frontal gyrus; ORBinf.R, orbital part of right inferior frontal gyrus; MFG.R, right middle frontal gyrus; REC.L, left rectus; ITG.R, right inferior temporal gyrus; PCUN.L, left precuneus; Cerebelum.R, right Cerebellum; IFGtriang.L, triangular part of left inferior frontal gyrus; IFGtriang.R, triangular part of right inferior frontal gyrus; PUT.L, left putamen; IPL.L, left inferior parietal lobule; PreCG.L, left precentral gyrus; ORBsup.L, orbital part of left superior frontal gyrus; aMPFC.L, left anterior medial prefrontal gyrus; vcMPFC.L, left ventral caudal medial prefrontal gyrus.


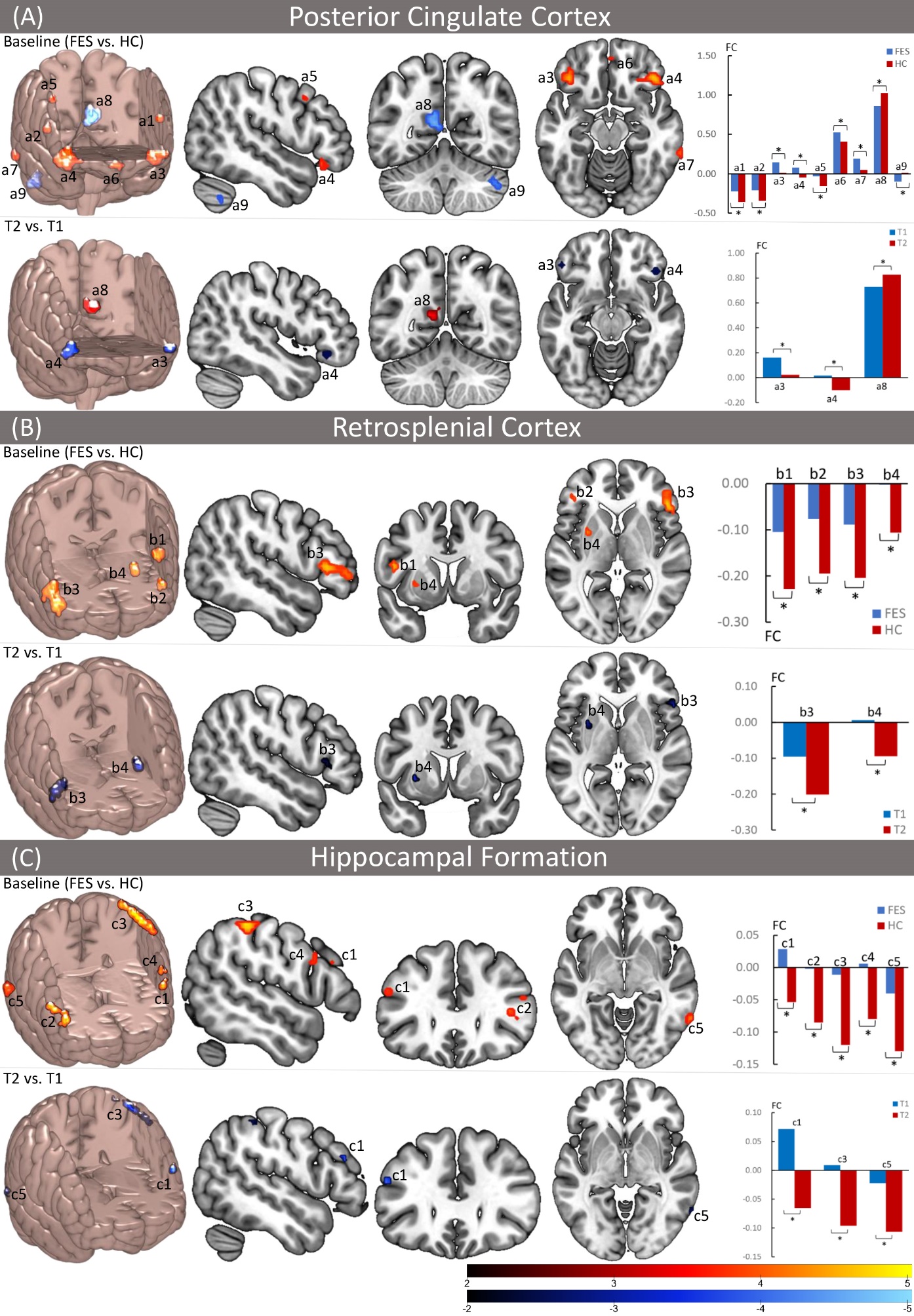


**Figure S1** Abnormal functional connectivity of the DMN at baseline and effect of long-term antipsychotic treatment on the DMN function in patients with more than 30% reduction of SAPS total scores. The left upper part in each panel depicted the brain regions with significantly increased (red) and/or decreased (blue) functional connectivity (FC) with the DMN seed regions (posterior cingulate cortex, retrosplenial cortex and hippocampal formation) at baseline in patients compared to healthy controls (*p* < .05, corrected by FDR), while the bar charts in the right upper part of each panel showed the mean values of the increased (red) and/or decreased (blue) FC with the DMN seed regions at baseline in patients and healthy controls; The left lower part brain maps in each panel depicted the brain regions with significant FC changes with the DMN seed regions after treatment in patients compared to their baseline (T2 vs. T1, *p* < .05, corrected by FDR), while the bar charts in the right lower part of each panel showed the mean values of the FC with significant changes after treatment both at baseline and follow-up time points. FES, first-episode schizophrenia patients; HC, healthy controls; T1, timepoint at baseline; T2, timepoint after treatment; FC, functional connectivity values; * means *p* < .05 (corrected by FDR); a1=opercular part of left inferior frontal gyrus, a2=opercular part of right inferior frontal gyrus, a3=orbital part of left inferior frontal gyrus, a4=orbital part of right inferior frontal gyrus, a5=right middle frontal gyrus, a6=left rectus gyrus, a7=right inferior temporal gyrus, a8=left precuneus, a9=right cerebellum; b1=opercular part of left inferior frontal gyrus, b2=triangular part of left inferior frontal gyrus, b3=triangular part of right inferior frontal gyrus, b4=left putamen; c1=triangular part of left inferior frontal gyrus, c2=triangular part of right inferior frontal gyrus, c3=left inferior parietal lobule, c4=left precentral gyrus, c5=right inferior temporal gyrus.

**Table S5** Treatment effect on DMN function in patients with more than 30% reduction of SAPS total scores

| ROI: Posterior Cingulate Cortex | | | | | |
| --- | --- | --- | --- | --- | --- |
| **Brain region** | **MNI** | | | **T Value** | **Voxels** |
|  | **x** | **y** | **z** |  |  |
| **T2 vs. T1** |  |  |  |  |  |
| ORBinf.L | -51 | 39 | -9 | -3.30 | 9 |
| ORBinf.R | 39 | 30 | -9 | -3.25 | 30 |
| PCUN.L | -15 | -57 | 15 | 3.69 | 24 |
| ROI: Retrosplenial Cortex | | | | | |
| **Brain region** | **MNI** | | | **T Value** | **Voxels** |
|  | **x** | **y** | **z** |  |  |
| **T2 vs. T1** |  |  |  |  |  |
| IFGtriang.R | 54 | 21 | -3 | -3.59 | 24 |
| PUT.L | -30 | 6 | 3 | -3.62 | 11 |
| ROI: Hippocampal Formation | | | | | |
| **Brain region** | **MNI** | | | **T Value** | **Voxels** |
|  | **x** | **y** | **z** |  |  |
| **T2 vs. T1** |  |  |  |  |  |
| IFGtriang.L | -51 | 30 | 24 | -4.09 | 6 |
| IPL.L | -63 | -36 | 39 | -3.08 | 58 |
| ITG.R | 63 | -60 | -3 | -2.80 | 10 |

**Note:** ROI, regions of interest (DMN subregions); MNI, Montreal Neurological Institute coordinate; T1, timepoint at baseline; T2, timepoint after treatment; ORBinf.L, orbital part of left inferior frontal gyrus; ORBinf.R, orbital part of right inferior frontal gyrus; PCUN.L, left precuneus; IFGtriang.R, triangular part of right inferior frontal gyrus; PUT.L, left putamen; IFGtriang.L, triangular part of left inferior frontal gyrus; IPL.L, left inferior parietal lobule; ITG.R, right inferior temporal gyrus.


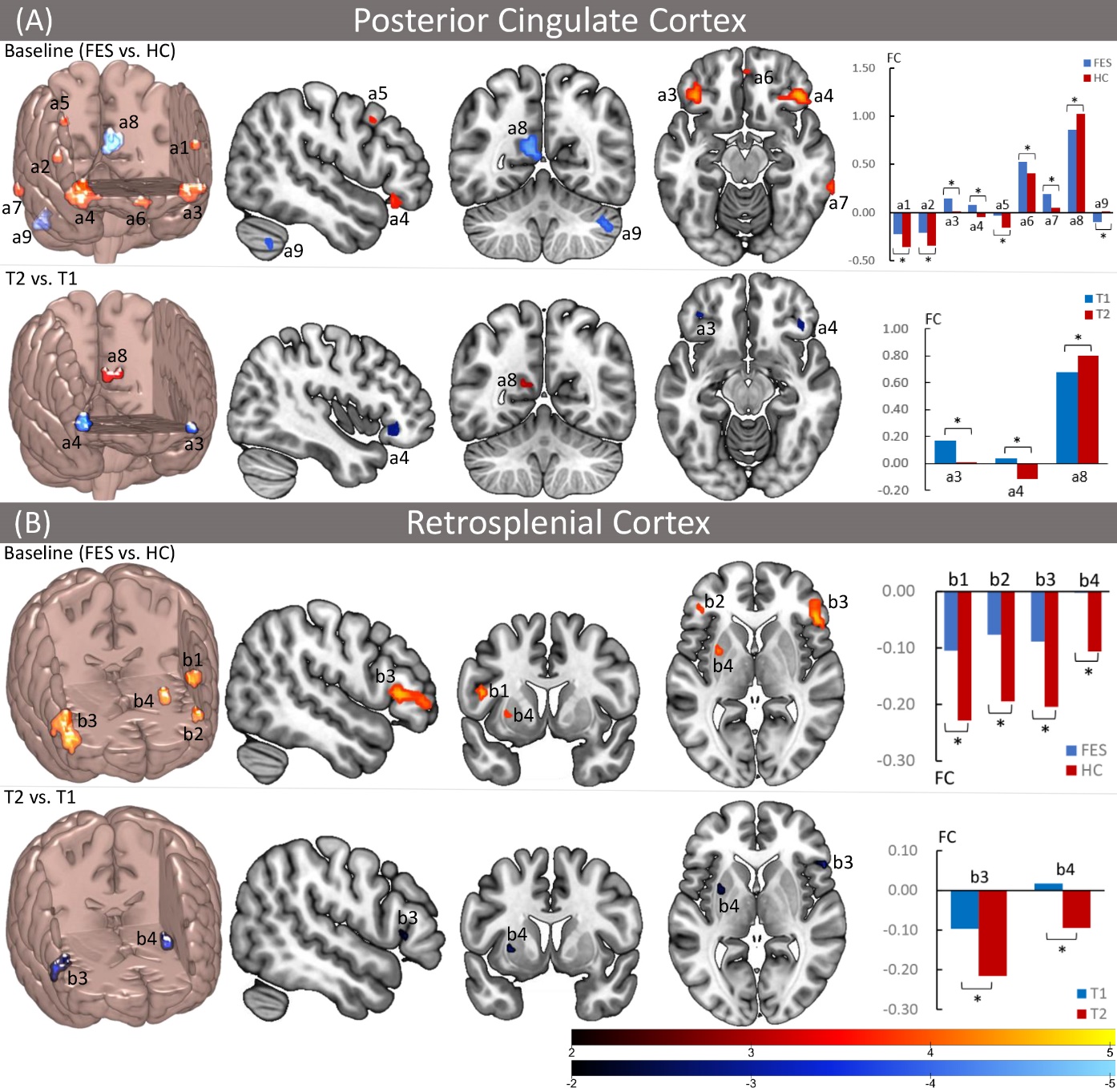


**Figure S2** Abnormal functional connectivity of the DMN at baseline and effect of long-term antipsychotic treatment on the DMN function in patients with treatment longer than 1year. The left upper part in each panel depicted the brain regions with significantly increased (red) and/or decreased (blue) functional connectivity (FC) with the DMN seed regions (posterior cingulate cortex and retrosplenial cortex) at baseline in patients compared to healthy controls (*p* < .05, corrected by FDR), while the bar charts in the right upper part of each panel showed the mean values of the increased (red) and/or decreased (blue) FC with the DMN seed regions at baseline in patients and healthy controls; The left lower part brain maps in each panel depicted the brain regions with significant FC changes with the DMN seed regions after treatment in patients compared to their baseline (T2 vs. T1, *p* < .05, corrected by FDR), while the bar charts in the right lower part of each panel showed the mean values of the FC with significant changes after treatment both at baseline and follow-up time points. FES, first-episode schizophrenia patients; HC, healthy controls; T1, timepoint at baseline; T2, timepoint after treatment; FC, functional connectivity values; * means *p* < .05 (corrected by FDR); a1=opercular part of left inferior frontal gyrus, a2=opercular part of right inferior frontal gyrus, a3=orbital part of left inferior frontal gyrus, a4=orbital part of right inferior frontal gyrus, a5=right middle frontal gyrus, a6=left rectus gyrus, a7=right inferior temporal gyrus, a8=left precuneus, a9=right cerebellum; b1=opercular part of left inferior frontal gyrus, b2=triangular part of left inferior frontal gyrus, b3=triangular part of right inferior frontal gyrus, b4=left putamen.

**Table S6** One year interval atypical antipsychotics treatment effect on DMN function in FES

| ROI: Posterior Cingulate Cortex | | | | | |
| --- | --- | --- | --- | --- | --- |
| **Brain region** | **MNI** | | | **T Value** | **Voxels** |
|  | **x** | **y** | **z** |  |  |
| **T2 vs. T1** |  |  |  |  |  |
| ORBinf.L | -39 | 42 | -15 | -4.55 | 5 |
| ORBinf.R | 39 | 33 | -9 | -4.40 | 23 |
| PCUN.L | -9 | -63 | 15 | 3.58 | 15 |
| ROI: Retrosplenial Cortex | | | | | |
| **Brain region** | **MNI** | | | **T Value** | **Voxels** |
|  | **x** | **y** | **z** |  |  |
| **T2 vs. T1** |  |  |  |  |  |
| IFGtriang.R | 54 | 21 | 3 | -3.66 | 13 |
| PUT.L | -27 | 6 | 0 | -2.98 | 10 |

**Note:** ROI, regions of interest (DMN subregions); MNI, Montreal Neurological Institute coordinate; T1, timepoint at baseline; T2, timepoint after treatment; ORBinf.L, orbital part of left inferior frontal gyrus; ORBinf.R, orbital part of right inferior frontal gyrus; PCUN.L, left precuneus; IFGtriang.R, triangular part of right inferior frontal gyrus; PUT.L, left putamen.


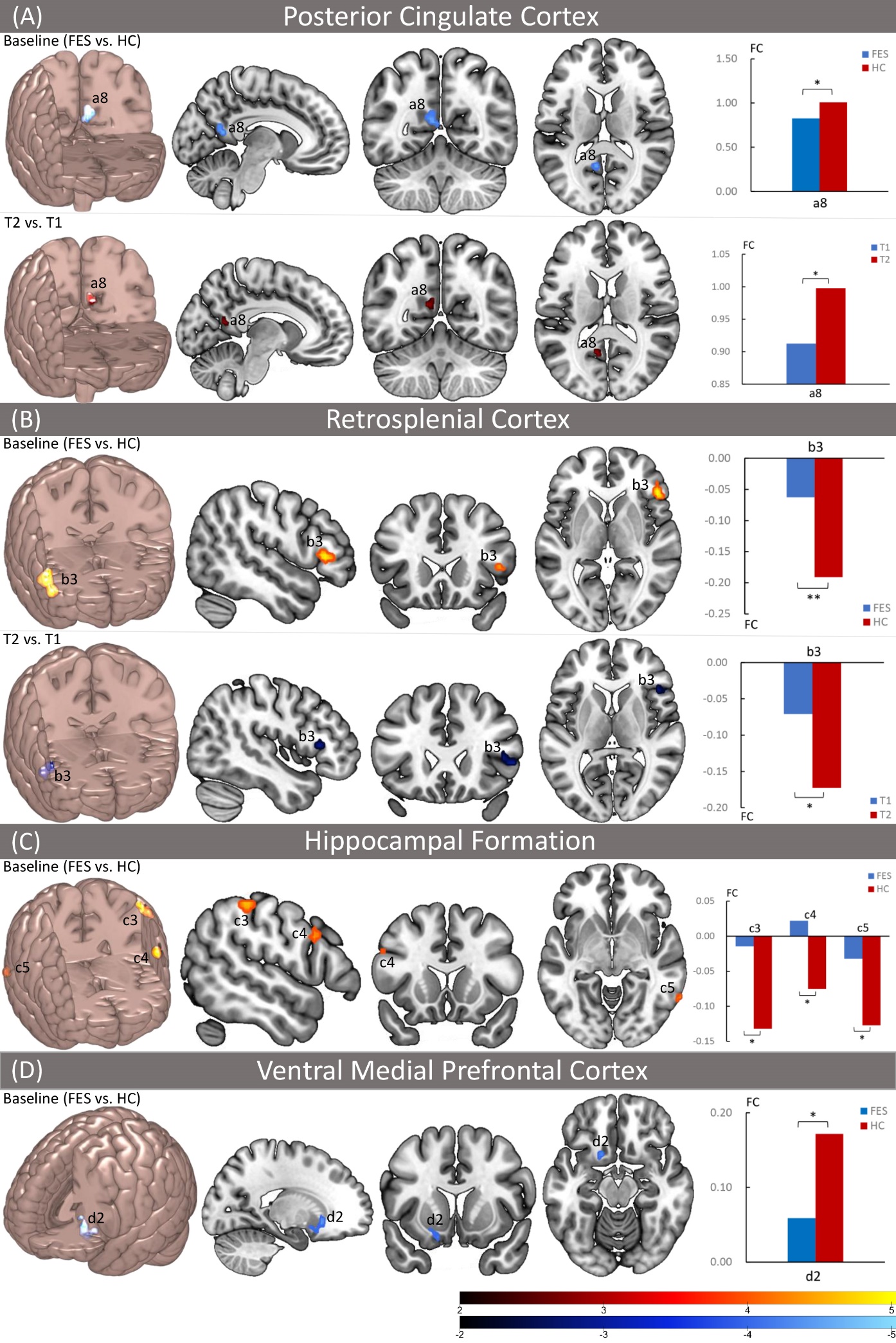


**Figure S3** Abnormal functional connectivity of the DMN at baseline and the effect of long-term antipsychotic treatment on the DMN function in FES patients (age≥16 years old). The left upper part of (A) and (B) as well as the left part of (C) and (D) depicted the brain regions with significantly increased (red) and/or decreased (blue) functional connectivity (FC) with DMN seed regions (posterior cingulate cortex, retrosplenial cortex, hippocampal formation and the ventral medial prefrontal cortex) at baseline in FES patients compared to healthy controls (*p* < .05, corrected by FDR), while the bar charts in the right upper part of (A) and (B) as well as the right part of (C) and (D) showed the mean values of the increased (red) and/or decreased (blue) FC with DMN seed regions at baseline in patients and healthy controls; The left lower part of brain maps in (A) and (B) depicted the brain regions with significant FC changes with DMN seed regions after treatment in FES patients compared to their baseline (T2 vs. T1, *p* < .05, corrected by FDR), while the bar charts in the right lower part of (A) and (B) showed the mean values of the FC with significant changes after treatment both at baseline and follow-up time points. FES, first-episode schizophrenia patients; HC, healthy controls; T1, time point at baseline; T2, time point after treatment; FC, functional connectivity values; * *p* < .05, ** *p* < .01 (corrected by FDR); a8=left precuneus, b3=triangular part of right inferior frontal gyrus, c3=left inferior parietal lobule, c4=left precentral gyrus, c5=right inferior temporal gyrus, d2=left ventral caudal medial prefrontal gyrus.

**Table S7** Abnormal functional connectivity of DMN in FES patients at baseline compared to healthy controls (age≥16 years old)

| ROI: Posterior Cingulate Cortex | | | | | |
| --- | --- | --- | --- | --- | --- |
| **Brain Region** | **MNI** | | | **T Value** | **Voxels** |
|  | **x** | **y** | **z** |  |  |
| **FES vs. HC** |  |  |  |  |  |
| PCUN.L | -6 | -57 | 12 | -4.62 | 26 |
| ROI: Retrosplenial Cortex | | | | | |
| **Brain Region** | **MNI** | | | **T Value** | **Voxels** |
|  | **x** | **y** | **z** |  |  |
| **FES vs. HC** |  |  |  |  |  |
| IFGtriang.R | 48 | 27 | 6 | 5.26 | 66 |
| ROI: Hippocampal Formation | | | | | |
| **Brain Region** | **MNI** | | | **T Value** | **Voxels** |
|  | **x** | **y** | **z** |  |  |
| **FES vs. HC** |  |  |  |  |  |
| IPL.L | -60 | -36 | 48 | 4.76 | 29 |
| PreCG.L | -54 | 12 | 30 | 4.43 | 14 |
| ITG.R | 66 | -57 | -6 | 4.44 | 9 |
| ROI: Ventral Medial Prefrontal Cortex | | | | | |
| **Brain Region** | **MNI** | | | **T Value** | **Voxels** |
|  | **x** | **y** | **z** |  |  |
| **FES vs. HC** |  |  |  |  |  |
| vcMPFC.L | -15 | 12 | -18 | -4.67 | 87 |

**Note:** ROI, regions of interest (DMN subregions); MNI, Montreal Neurological Institute coordinate; FES, first-episode schizophrenia patients; HC, healthy controls; PCUN.L, left precuneus; IFGtriang.R, triangular part of right inferior frontal gyrus; IPL.L, left inferior parietal lobule; PreCG.L, left precentral gyrus; ITG.R, right inferior temporal gyrus; vcMPFC.L, left ventral caudal medial prefrontal gyrus.

**Table S8** Effect of long-term atypical antipsychotic treatment on DMN function in FES (age≥16 years old)

| ROI: Posterior Cingulate Cortex | | | | | |
| --- | --- | --- | --- | --- | --- |
| **Brain Region** | **MNI** | | | **T Value** | **Voxels** |
|  | **x** | **y** | **z** |  |  |
| **T2 vs. T1** |  |  |  |  |  |
| PCUN.L | -9 | -60 | 15 | 2.91 | 20 |
| ROI: Retrosplenial Cortex | | | | | |
| **Brain Region** | **MNI** | | | **T Value** | **Voxels** |
|  | **x** | **y** | **z** |  |  |
| **T2 vs. T1** |  |  |  |  |  |
| IFGtriang.R | 54 | 24 | 3 | -3.15 | 21 |

**Note:** ROI, regions of interest (DMN subregions); MNI, Montreal Neurological Institute coordinate; T1, timepoint at baseline; T2, timepoint after treatment; PCUN.L, left precuneus; IFGtriang.R, triangular part of right inferior frontal gyrus.

**References:**

Power JD, Barnes KA, Snyder AZ, et al. (2012) Spurious but systematic correlations in functional connectivity MRI networks arise from subject motion. *Neuroimage* 59: 2142-2154.
